# A novel lncRNA, LL22NC03-N64E9.1, represses KLF2 transcription through binding with EZH2 in colorectal cancer

**DOI:** 10.18632/oncotarget.19738

**Published:** 2017-07-31

**Authors:** Yifan Lian, Changsheng Yan, Jie Ding, Rui Xia, Zhonghua Ma, Bingqing Hui, Hao Ji, Jing Zhou, Keming Wang

**Affiliations:** ^1^ The Second Clinical Medical College of Nanjing Medical University, Nanjing 210000, Jiangsu, People's Republic of China; ^2^ Department of Gastroenterology, Zhongshan Hospital Affiliated to Xiamen University, Xiamen 361004, Fujian, People's Republic of China; ^3^ Department of Obstetrics and Gynecology, The First Affiliated Hospital of Nanjing Medical University, Nanjing, 210000, Jiangsu, People's Republic of China; ^4^ Department of Oncology, Second Affiliated Hospital, Nanjing Medical University, Nanjing 210000, Jiangsu, People's Republic of China; ^5^ Department of Biochemistry and Molecular Biology, Nanjing Medical University, Nanjing, 210000 Jiangsu, People's Republic of China

**Keywords:** colorectal cancer, proliferation, LL22NC03-N64E9.1, KLF2, lncRNA

## Abstract

Long noncoding RNAs (lncRNA) have been implicated in variety human cancer but their mechanisms of function are mainly undocumented. In the present study, we investigated lncRNAs alteration that contributed to colorectal cancer (CRC) by utilizing TCGA RNA sequencing data and other publicly available lncRNAs expression profiling data. Here, We screened out the CRC–associated lncRNA LL22NC03-N64E9.1, a key regulator of CRC development and progression. We also revealed that knockdown of LL22NC03-N64E9.1 inhibited cell proliferation, colony formation, tumorigenicity and apoptosis promotion, both *in vitro* and *in vivo*. Mechanistically, LL22NC03-N64E9.1 repressed underlying target gene KLF2 transcription through binding to EZH2. Furthermore, rescue experiments revealed that LL22NC03-N64E9.1 oncogenic function may partially depend on repressing KLF2. Taken together, our results suggested that LL22NC03-N64E9.1 confered an oncogenic function in human CRC and may serve as a candidate prognostic biomarker and target for new therapies in this deadly disease.

## INTRODUCTION

Colorectal cancer (CRC), one of the most common types of malignant worldwide, is the second leading cause of cancer-related mortality in the Western countries [1, 2]. Almost 90% of CRC patients can be cured if detected at an early stage. Unfortunately, patients with CRC are very often diagnosed at advanced stages with poor prognosis [3, 4]. Generally, the tumorigenicity of CRC is a multistep process involving a number of genetic or epigenetic alterations that eventually result in malignant transformation of CRC cells [5, 6]. Therefore, better understanding of the molecular mechanisms involved in the development and progression of CRC is essential for the development of diagnostic bio-markers and potential therapeutic targets for CRC patients [7].

Over the past decade, complement of human genome sequencing and GENCODE project has showed that only about 2% of human genome are protein coding genes, while the major of the rest is non-coding genes yielding lots of non-coding transcripts including miRNAs, circular RNA and long non-coding RNAs (lncRNAs) [8]. Among these, lncRNAs represent a group of non-coding RNAs of more than 200 nucleotides in length and have no protein-coding capacity [9]. Recently, accumulating evidence revealed that a subset of these non-coding RNAs are functional and they have myriad molecular functions through many cellular pathways and processes, including oncogenic or tumor suppress signaling [10]. For instance, lncRNA HOXA11-AS is highly expressed in gastric cancer, promoting cell proliferation and invasion via scaffolding the chromatin modification factors PRC2, LSD1 and DNMT1 [11]. We previously found that HOTTIP promotes CRC cell proliferation partly via down-regualtion of p21 expression, while IRAIN could promote pancreatic cancer cell growth by binding to histone demethylase lysine-specific demethylase 1 (LSD1) and enhancer of zeste homolog 2 (EZH2) [12, 13]. Therefore, there is a closely link between lncRNAs and cancer.

KLF2 is one of the key members in the Kruppel-like factor (KLF) family due to its tumor suppressor function in malignancies such as breast cancer, non-small cell lung cancer, prostate cancer and leukemia [14–18]. It was reported that KLF2 could inhibit tumor cell proliferation mediated by KRAS [18]. Moreover, previous study showed that EZH2 could directly bind to KLF2 promoterto silence KLF2 expression, resulting in the block of the tumor-suppressor features of KLF2 [19].

In our present study, we analyzed TCGA colorectal cancer and normal tissue RNA sequencing data and two independent microarray data sets from Gene Expression Omnibus and identified a novel CRC-associated lncRNA LL22NC03-N64E9.1. The LL22NC03-N64E9.1 gene is located at chromosomal locus 22q11.1 and transcribes a 1388nt transcript. By binding to EZH2, a core component of the polycomb repressive complex 2 (PRC2), LL22NC03-N64E9.1 epigenetically suppressed KLF2 expression via histone modification. The silence of LL22NC03-N64E9.1 induced CRC cell cycle arrest and significantly inhibited tumor growth both *in vivo* and *in vitro*. Our results indicated that LL22NC03-N64E9.1 may serve as a candidate target for new therapies in human colorectal cancer.

## RESULTS

### LL22NC03-N64E9.1 is upregulated in human CRC tissues and is positively correlated with larger tumor size and advanced TNM stage

**Figure 1:**
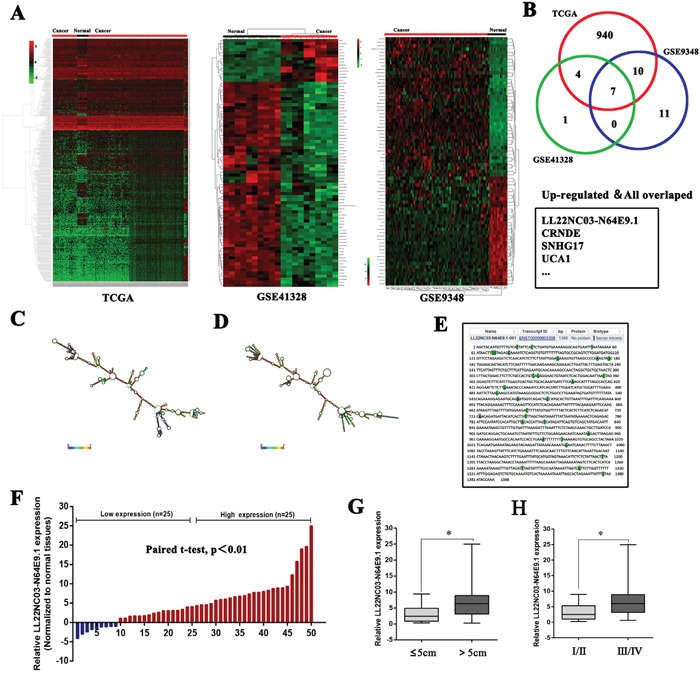
Relative expression of LL22NC03-N64E9.1 in CRC tissues and its clinical significance **(A)** Hierarchical clustering analysis of lncRNAs that were differentially expressed (fold change > 2; P < 0.05) in colorectal cancer and normal tissues. **(B)** Overlap of dysregulated lncRNAs in TCGA data and GEO datasets. **(C, D)** Prediction of LL22NC03-N64E9.1 structure based on minimum free energy (MFE) and partition function. Color scale indicates the confidence for the prediction for each base with shades of red indicating strong confidence. (http://rna.tbi.univie.ac.at/). **(E)** The full sequence of LL22NC03-N64E9.1 was published in Ensembl database (ENST00000603308.1). **(F)** Relative expression of LL22NC03-N64E9.1 in CRC tissues compared with corresponding adjacent normal tissues (n=50), and LL22NC03-N64E9.1 expression was classifid into two groups. **(G, H)** LL22NC03-N64E9.1 expression was significantly higher in patients with a larger tumor size and a higher pathological stage (shown as ΔCT). Bars: s.d., *P<0.05, **P<0.01.

To identify the lncRNAs that are involved in colorectal tumorigenesis, we performed an integrative analysis of TCGA colorectal cancer and normal tissue RNA sequencing data and CRC microarray profile comprising GSE9348 and GSE21510 from GEO datasets. We identified 961 lncRNAs that was upregulated in the TCGA data, 28 in GSE9348 datasets, and 12 in GSE41328 datasets (fold change > 2.0, P < 0.05; Figure 1A;) but only seven lncRNAs consistently upregulated in all datasets (Figure 1B). To prioritize the most CRC biologically relevant lncRNAs, we focused on lncRNA LL22NC03-N64E9.1 which is most highly expressed in colorectal cancer tissues. In addition, LL22NC03-N64E9.1 is a long non-coding RNA without any functional report yet. Thus, We selected LL22NC03-N64E9.1 for further investigation. The LL22NC03-N64E9.1 gene is located at the chromosomal locus 22q11.1 and encodes a 1388bp transcript (Figure 1E). Next, to validate the analysis results, we detected LL22NC03-N64E9.1 expression in an cohort of 50 pair colorectal cancer and normal tissues. The results confirmed that LL22NC03-N64E9.1 expression is increased in colorectal cancer tissues compared with the adjacent normal tissues (Figure 1F). To further investigated the relationship between LL22NC03-N64E9.1 expression and clinical pathological features, we divided the samples into high (above the median, n=25) and low (below the median, n=25) LL22NC03-N64E9.1 expression groups according to the median value of all CRC samples (Figure 1F). A chi-square test was then performed to evaluate the clinicopathological features between the two groups. As shown in Table 1, increased LL22NC03-N64E9.1 in CRC tissues were significantly correlated with larger tumor sizes (P<0.05) and advanced TNM stages (P<0.05) (Figure 1G and 1H). However, several other clinical parameters were found to be less significantly associated with LL22NC03-N64E9.1expression (Table 1)

**Table 1:** Correlation between LL22NC03-N64E9.1 expression and clinicopathologic characteristics of patients with CRC.(n=50)

Characteristics	LL22NC03-N64E9.1		*P*
	Low No. Cases(n=25)	High No. Cases(n=25)	Chi-squared test, *P*-value
**Age(years)**			0.551
≤60	7	10	
>60	18	15	
**Gender**			1.00
Male	16	15	
Female	9	10	
**Tumor size(cm)**			0.022
≤5	16	7	
>5	9	18	
**TNM Stage**			0.010
I/II	16	6	
III/IV	9	19	
**Distant metastasis**			0.138
Positive	2	7	
Negative	23	18	

### Knockdown of LL22NC03-N64E9.1 inhibits CRC cell proliferation, induces apoptosis and promotes cell cycle arrest

**Figure 2:**
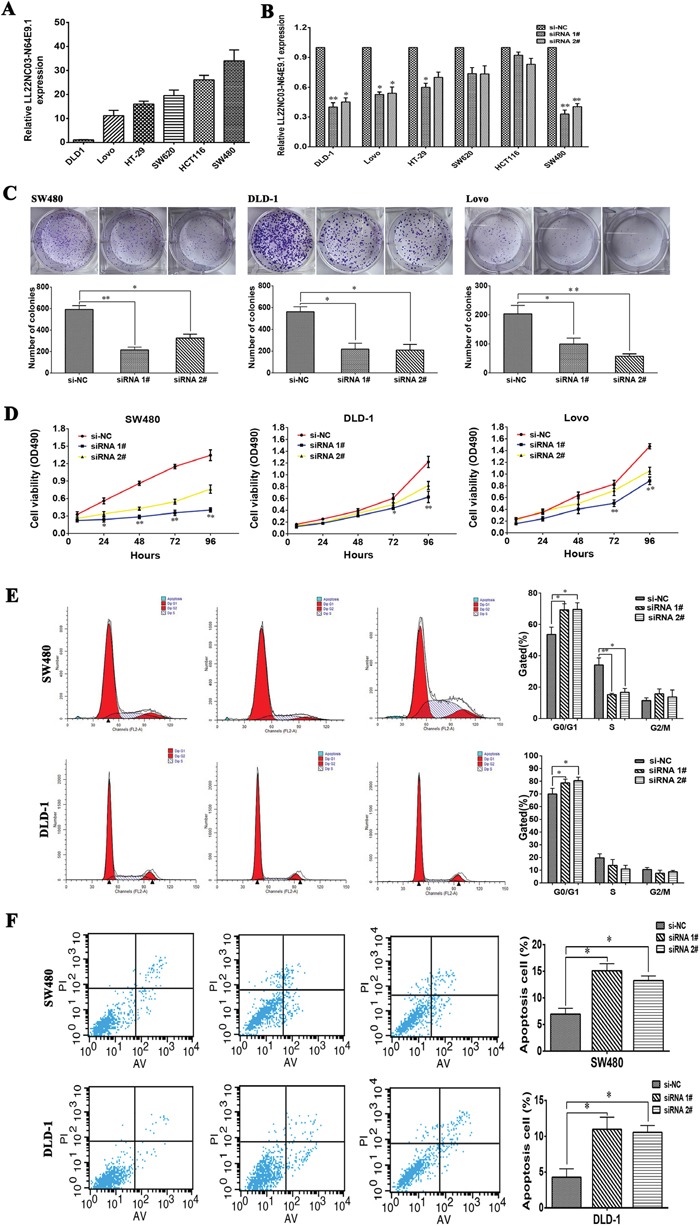
Effects of LL22NC03-N64E9.1 on CRC proliferation and apoptosis *in vitro* **(A)** Analysis of LL22NC03-N64E9.1 expression levels in CRC cell lines (DLD-1, Lovo, HT-29, SW620, HCT116 and SW480) by qRT-PCR. **(B)** LL22NC03-N64E9.1 knockdown in CRC cells transfected with siRNA. **(C)** Representative results of the colony formation of SW480, DLD-1 and Lovo cells transfected with the siRNA of LL22NC03-N64E9.1. **(D)** MTT assays were performed to determine the cell viability of CRC cells after the transfection of siRNA against LL22NC03-N64E9.1. **(E)** Flow cytometry assays were performed to analysis the cell cycle progression when SW480 and DLD-1 cells transfected with siRNA against LL22NC03-N64E9.1. **(F)** Flow cytometry assays were performed to analysis the cell apoptotic in siRNA-transfected SW480 and DLD-1 cells. Representative images and data based on three independent experiments. Bars: s.d, *P<0.05, **P<0.01.

To further investigate the biological role of LL22NC03-N64E9.1 in CRC, LL22NC03-N64E9.1 expression was detected in the CRC cell lines, including DLD-1, Lovo, HT-29, HCT116, SW620 and SW480. Significantly high LL22NC03-N64E9.1 expression was found in SW480, HCT116, SW620, HT-29 and Lovo compared with that in DLD-1(Figure 2A). Then, two LL22NC03-N64E9.1 siRNAs were transfected into six CRC cell lines, respectively. The qRT-PCR results revealed that LL22NC03-N64E9.1 was sufficiently silenced in three CRC cell lines, including SW480, DLD-1 and Lovo (Figure 2B). Compared with the negative control (NC) siRNA, siRNAs targeting LL22NC03-N64E9.1 significantly inhibited cell proliferation ability in CRC cell lines based on the colony formation assay (Figure 2C). Consistent with the results of the colony formation assay, cell viability was inhibited after transfection with siRNA in the MTT assay (Figure 2D). To investigate whether LL22NC03-N64E9.1 influence the cell cycle distribution of SW480 and DLD-1 as cells *in vitro*, flow cytometry was used to detect the percentage of cells in G0/G1, S, and G2/M. The results revealed that siRNA-LL22NC03-N64E9.1 induced significant G0-G1 phase arrest and decreased the percentage of cells in the S phase (Figure 2E). To further determine whether the effect of LL22NC03-N64E9.1 on CRC cells proliferation reflected cell apoptosis, we performed flow cytometry assays. The results showed that SW480 and DLD-1 cells transfected with LL22NC03-N64E9.1 siRNA 1# or siRNA 2# had higher apoptotic rate compared to the control (Figure 2F). These data demonstrated that LL22NC03-N64E9.1 could promote the proliferation phenotype and inhibit apoptosis of colorectal cancer cells.

### Knockdown of LL22NC03-N64E9.1 inhibits CRC cell tumorigenesis *in vivo*

**Figure 3:**
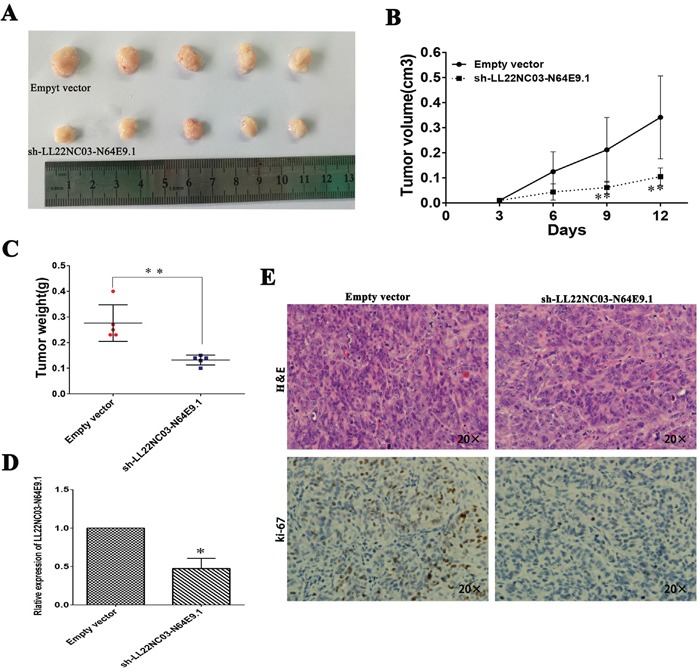
The silencing of LL22NC03-N64E9.1 inhibited CRC growth *in vivo* **(A)** The stable LL22NC03-N64E9.1 knockdown SW480 cells were used for the *in vivo* study. The nude mice carrying tumors from respective groups were shown. **(B)** Tumor volumes were calculated after injection every 3 days. **(C)** Tumor weights from two groups are represented. **(D)** qRT-PCR was performed to detect the average expression of LL22NC03-N64E9.1 in xenograft tumors (n = 5). **(E)** Images of HE staining and immunohistochemistry of the xenograft tumors. Representative Ki-67 protein levels in xenograft tumors as evaluated by IHC. Representative images and data based on three independent experiments. Bars: s.d, *P<0.05, **P<0.01.

To confirm the impact of LL22NC03-N64E9.1 on CRC cell growth *in vivo*, SW480 cells transfected with sh-LL22NC03-N64E9.1 or empty vector were injected into male nude mice while the cells were transfected with empty vector served as the control. At 12 days post-injection, the tumor growth in the sh-LL22NC03-N64E9.1 group was markedly slower than that in the control group (Figure 3A). Correspondingly, the tumor volumes and weights were obviously decreased compared with the controls (Figure 3B, 3C). As shown, qRT-PCR confirmed that the LL22NC03-N64E9.1 expression level was lower in the tumor tissues derived from the sh-LL22NC03-N64E9.1-transfected cells (Figure 3D). Moreover, immunohistochemistry (IHC) analysis confirmed that the tumors formed from SW480/sh-LL22NC03-N64E9.1 cells displayed weaker Ki-67 staining than those formed from the control cells (Figure 3E). Our results indicated that silenced LL22NC03-N64E9.1 expression could suppress colorectal cancer cells tumor growth *in vivo*.

### LL22NC03-N64E9.1 inhibits KLF2 expression by binding to EZH2

**Figure 4:**
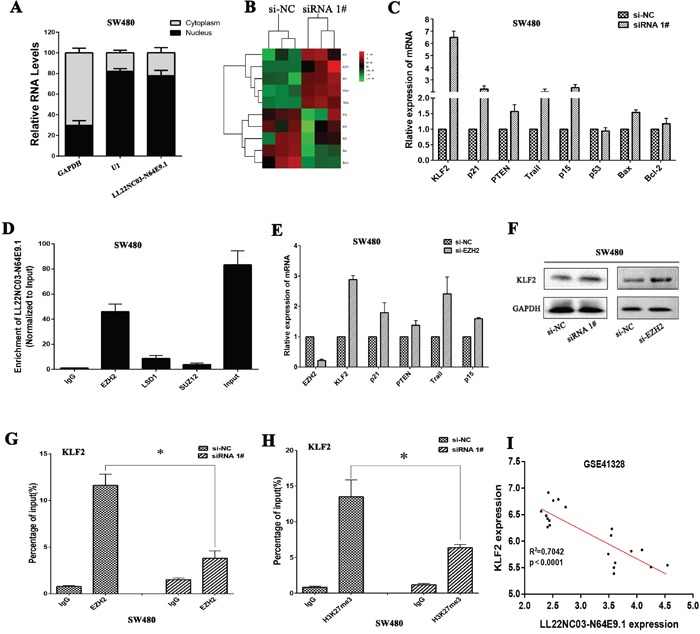
LL22NC03-N64E9.1 epigenetically silences KLF2 transcription by binding with EZH2 **(A)** Relative LL22NC03-N64E9.1 levels in cell cytoplasm or nucleus of SW480 cell lines were detected by qRT-PCR. **(B)** Heat map of altered genes in LL22NC03-N64E9.1 knockdown SW480 cells compared with control cells. **(C)** The levels of KLF2, p21, PTEN, Trail, p15, p53, Bax and Bcl-2 mRNA were detected by qRT-PCR when knockdown of LL22NC03-N64E9.1 in SW480 cells. **(D)** RIP assays were performed in SW480 cells and the coprecipitated RNA was subjected to qRT-PCR for LL22NC03-N64E9.1. **(E)** The expression of EZH2, KLF2, p21, PTEN, Trail and p15 in SW480 cells, after knockdown of EZH2. **(F)** The KLF2 protein levels were determined by western blot in LL22NC03-N64E9.1 or EZH2 knockdown SW480 cells. **(G, H)** ChIP-qPCR of H3K27me3 and EZH2 of the promoter region of the KLF2 locus after siRNA treatment targeting si-NC or si-LL22NC03-N64E9.1 in SW480 cells, **(I)** The relationship between LL22NC03-N64E9.1 expression and KLF2 mRNA levels was analyzed in the profile of CRC patient tissue from Gene Expression Omnibus (GEO). Representative images and data based on three independent experiments. Bars: s.d, *P<0.05, **P<0.01.

To investigate the potential molecular mechanism of LL22NC03-N64E9.1 in CRC cells, we firstly analysis the distribution of LL22NC03-N64E9.1 in SW480 cells and we found that LL22NC03-N64E9.1 was mostly located in nucleus (Figure 4A). A Heat map showed expression levels of 10 different cell cycle related transcripts in SW480 cells with knockdown of LL22NC03-N64E9.1 expression for 48 hours (Figure 4B). Several genes that may contribute to CRC were selected and confirmed by qRT-PCR assays (Figure 4C). Among these altered genes, KLF2 has been identified as a novel tumor suppressor involved in cancer cell proliferation and apoptosis. Therefore, we chose KLF2 for further investigation. Previously studies have revealed that lncRNAs contribute to tumor cells phenotype through inhibiting tumor suppressors or activation of oncogenes via interacting with specific RNA binding proteins [20, 21]. In addition, several studies have indicated that approximately 24% of lncRNAs can regulate downstream target genes by binding with Polycomb repressive complexe 2 (PRC2) [22, 23]. Given this background, we predicted the interaction probabilities of LL22NC03-N64E9.1 and RNA binding proteins via RNA-protein interaction prediction (http://pridb.gdcb.iastate.edu/RPISeq/), and found that LL22NC03-N64E9.1 potentially binds EZH2, LSD1, and SUZ12 (as the RF or SVM score > 0.5). We next performed RIP assays and confirmed that LL22NC03-N64E9.1 could interact with EZH2 but not other RNA binding proteins in SW480 cells (Figure 4D). We further investigated the functional relevance of the interaction between EZH2 and LL22NC03-N64E9.1. EZH2 was first knockdown using siRNA, and significant upregulation of KLF2 was observed (Figure 4E). Westernblot analysis also confirmed that knockdown of LL22NC03-N64E9.1 or EZH2 could upregulate KLF2 in SW480 cells (Figure 4F). Current evidence has demonstrated that the PRC2 complex is a negative regulator of transcription via the trimethylation of histone 3 lysine 27 (H3K27me3) [24]. Thus, it is very likely that LL22NC03-N64E9.1 suppresses KLF2 expression by recruiting the PRC2 complex to KLF2 promoter region, leading to trimethylation of H3K27 at this region. Next, We designed three paired primers across the promoter region (2000bp) of KLF2, and then conducted chromatin immunoprecipitation (ChIP) analysis by LL22NC03-N64E9.1 knockdown. The ChIP assays demonstrated that knockdown of LL22NC03-N64E9.1 decreased the binding of EZH2 and H3K27me3 levels across the KLF2 promoters (Figure 4G and 4H). Finally, we analyzed the correlation between LL22NC03-N64E9.1 and KLF2 expression in CRC patient profiles from GEO, and found that there was a significantly negative correlation (Figure 4I). Together, our finding suggested that LL22NC03-N64E9.1 could epigenetically suppress the expression of KLF2 by binding to EZH2 (one key components of PRC2), thus promoting CRC development.

### Silence of KLF2 is partly involved in the oncogenic function of LL22NC03-N64E9.1

**Figure 5:**
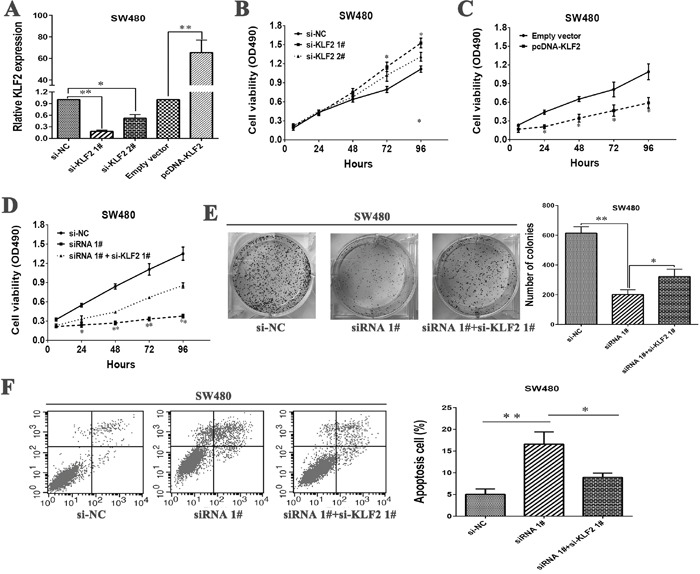
LL22NC03-N64E9.1 negatively regulates expression of KLF2 by rescue experiments **(A)** The levels of KLF2 mRNA expression were determined by qRT-PCR when SW480 cells were transfected with si-NC, si-KLF2 1#, si-KLF2 1# and Empty vector, pcDNA-KLF2. **(B)** MTT assays were performed to determine the cell viability of SW480 cells after the transfection of siRNA against KLF2. **(C)** MTT assays were performed to determine the viability of pcDNA-KLF2-transfected SW480 cells. **(D, E)** MTT and colony formation assays were used to determine the cell proliferation ability for SW480 cells transfected with si-NC and siRNA 1# and co-transfected with siRNA 1# and si-KLF2. **(F)** Flow cytometry assays were performed to analyze the cell apoptosis when SW480 cells were transfected with si-NC and siRNA 1# and co-transfected with siRNA 1# and si-KLF2. Representative images and data based on three independent experiments. Bars: s.d, *P<0.05, **P<0.01.

To validate the influence of KLF2 on cellular proliferation in CRC cells, KLF2 expression was knocked down or up-regulation in SW480 cells (Figure 5A). MTT assays was then conducted to detect the cell viability. The results revealed that knockdown KLF2 expression could promote cellular proliferation (Figure 5B). Whereas, stimulated KLF2 expression promoted cell proliferation in SW480 cells (Figure 5C). These data showed that KLF2 could inhibit cell proliferation of SW480 cells, which was contrary to results of downregulated LL22NC03-N64E9.1 in CRC cells.

Next, To further investigate whether KLF2 is involved in the LL22NC03-N64E9.1-induced increase of CRC cells apoptosis and cycle arrest, we performed rescue experiments. SW480 cells were co-transfected with si-LL22NC03-N64E9.1 and si-KLF2 1#. The results of MTT and colony formation assays indicated that the proliferation ability of SW480 cells co-transfected with si-LL22NC03-N64E9.1 and si-KLF2 1# were improved comparing with SW480 cells transfected with si-LL22NC03-N64E9.1 (Figure 5D and 5E). Furthermore, apoptosis rate also was rescued in co-transfected-group comparing with si-LL22NC03-N64E9.1 group (Figure 5F). These results indicate that LL22NC03-N64E9.1 exerts biological effect on CRC cells may partly via repressing KLF2 expression.

## DISCUSSION

In this study, we identified that the novel lncRNA LL22NC03-N64E9.1 is overexpression in human colorectal cancer tissue. In addition, upregulation of LL22NC03-N64E9.1 could promote CRC cells proliferation and inhibit cell apoptosis. Furthermore, LL22NC03-N64E9.1 could epigenetically inhibit the expression of KLF2 by binding to EZH2, thus promoting CRC progress.

Recent findings have suggested that many lncRNAs have important biological roles in CRC, such as CASC2 [25], UCA1 [26], CCAT2 [27] and HOTAIR [28] et al. Our previous work also identified that lncRNA Loc554202 induces CRC cell apoptosis through the activation of specific caspase cleavage cascades [29]. Therefore, identification of CRC-associated lncRNAs may provide a missing piece of the oncogenic and tumor suppressor network puzzle. In this study, we compared the lncRNA profiles of CRC and normal tissues using the sequencing data from TCGA project and microarray profile from GEO. We screened out a novel lncRNA, LL22NC03-N64E9.1, whose expression significantly increased. Next, we found that the expression of LL22NC03-N64E9.1 was upregulated in an cohort of 50 pair human CRC tissues compared with that in the corresponding normal tissues and correlated with CRC progression, although further studies with increased CRC tissue sample sizes and better patient characteristics are needed.

It has been shown that lncRNAs play an important roles in regulating the malignant phenotypes of cancer cells [30, 31]. Our data also showed that LL22NC03-N64E9.1 is capable of promoting CRC cell proliferation and inhibiting cell apoptosis. Generally, lncRNAs is involved in regulation of cancer cells phenotypes by regulating target gene expression with different molecular mechanisms, including chromatin modification, genomic imprinting, RNA decay, sponging miRNAs and binding with RBP [21, 32]. Previous studies found that lncRNA XIST was upregulated in non small cell lung cancer (NSCLC) and could promote cancer cell proliferation and invasion. They also found that KLF2 was one direct target of XIST. Here, we found that LL22NC03-N64E9.1 could directly bind to EZH2, then repress downstream target gene KLF2 expression. KLF2, a key member of the Kruppel-like factor (KLF) family that with Cys2/His2 zinc-finger domains, which is reported to exert tumor suppressor functions in many malignancies, but its functional role in CRC remains unclear [33, 34]. In this study, we found that KLF2 also functions as tumor suppressors in CRC cells and knockdown of KLF2 is involved in the LL22NC03-N64E9.1-exerted oncogenic function in CRC cells. Meanwhile, correlation analysis in CRC tissues showed that LL22NC03-N64E9.1 expression is negatively associated with KLF2 expression. Notably, it is highly possible that LL22NC03-N64E9.1 could regulate a set of other target genes and RNA-sequencing following LL22NC03-N64E9.1 knockdown may help to identify the downstream targets of LL22NC03-N64E9.1.

In summary, this is the first study to show that L22NC03-N64E9.1 is upregulated in CRC tissues and its overexpression may be associated with CRC progression. L22NC03-N64E9.1 could promote CRC cell proliferation and tumorigenesis partly via epigenetically silencing KLF2 transcription by binding to EZH2. L22NC03-N64E9.1 may serve as a target for new therapies in CRC.

## MATERIALS AND METHODS

### Expression profiling data retrieval and analysis of lncRNAs in colorectal cancer

CRC gene expression data were downloaded from the TCGA and GEO dataset. The independent data sets from GSE9348, and GSE41328 were analyzed in this study. The BAM files and normalized probe-level intensity files were downloaded from TCGA and GEO databases, respectively. The probe sequences were downloaded from GEO or microarray manufacturers, and bowtie was used to re-annotate probes according to GENCODE Release 19 annotation for lncRNAs. For multiple probes corresponding to one gene, the probe with the maximum signal was selected to generate expression of lncRNAs.

### Tissue collection and ethics statement

A total of 50 patients analyzed in this study underwent resection of the primary colorectal cancer at the Second Affiliated Hospital of Nanjing Medical University. All collected tissue samples were immediately snap frozen in liquid nitrogen and stored at −80°C before need. The study was approved by the Research Ethics Committee of Nanjing Medical University (Nanjing, Jiangsu, PR China), and written informed consent was obtained from all patients. The clinicopathological characteristics of the colorectal cancer patients are summarized in Table 1.

### Total RNA isolation and qRT-PCR assays

Total RNA was extracted from tissues or cultured cells using TRIzol reagent (Invitrogen, Carlsbad, CA, USA) according to the manufacturer's protocol. RNA quantity and quality were determined by NanoDrop2000c (Thermo Scientific, Waltham, MA, USA). For qRT-PCR, 1 μg of RNA was reverse transcribed to cDNA using a Reverse Transcription Kit (Takara, Dalian, China). The quantitative polymerase chain reaction (qRT-PCR) assays were conducted on an ABI 7500. Data were normalized to the expression of glyceralde-hyde-3-phosphatedehydrogenase (GAPDH). Primers used for target amplification are listed in Supplementary Table 1.

### Cell lines and culture conditions

Four CRC cell lines (DLD-1, Lovo, HT-29, SW480, SW620, and HCT116) were obtained from the American Type Culture Collection (Manassas, VA, USA). All of the cell lines were grown and maintained in RPMI 1640 Medium (Invitrogen) supplemented with 10% fetal bovine serum (FBS), 100 U/ml penicillin, and 100 mg/ml streptomycin (Invitrogen, Shanghai, China) at 37°C with 5% CO_2_.

### Cell transfection

Typically, CRC cells were seeded at six-well plates and then transfected in the next day with specific siRNA (100 nM) or control siRNA (100 nM) using Lipofectamine 2000 (Invitrogen), according to the manufacturer's protocol (Invitrogen). After transfection, the cells were harvested for further studies. The primer sequences and siRNA sequences are summarized in Supplementary Table 1.

### Cell viability and colony formation assay

Cell viability was monitored using the Cell Proliferation Reagent Kit I (MTT; Roche Applied Science). The DLD-1, SW480 and Lovo cells were transfected with siRNA or si-NC (3000 cells/well) and were cultured in 96-well plates with six replicate wells. Cell viability was assessed according to the manufacturer's recommendations. For the colony formation assay, a total of 500 cells were placed in a six-well plate and maintained in medium containing 10% FBS. The medium was replaced every 4 days. After 2 weeks, cells were fixed with methanol and stained with 0.1% crystal violet (SigmaAldrich). Visible colonies were manually counted. Triplicate wells were measured in each treatment group.

### Flow cytometry

DLD-1 and SW480 cells transfected with siRNA or si-NC were harvested after 48h. Subsequently, the cells were stained with PI using the CycleTESTTM PLUS DNA Reagent Kit (BD Biosciences) according to the protocol and analyzed with a flow cytometer (FACScan^®^;BDBiosciences) equipped with the CellQuest software (BD Biosciences). The percentages of the cells in G0-G1, S, and G2-M phases were calculated and compared. DLD-1 and SW480 cells transfected with siRNA or si-NC were harvested after 48h for apoptosis analysis. The cells were then treated with fluorescein isothiocyanate (FITC) Annexin V and propidium iodide (PI) in the dark at room temperature according to the manufacturer's recommendations. Subsequently, the cells were analyzed by FACScan^®^, and they were identified as viable, dead, early apoptotic, or late apoptotic cells.

### *In vivo* tumor formation assay

Four-week-old male athymic mice were purchased from the Animal Center of the Nanjing University (Nanjing, China) and maintained in pathogen-free conditions. SW480 cells were transfected with sh-LL22NC03-N64E9.1 or empty vector and harvested from six-well plates, washed with phosphate-buffered saline (PBS), and resuspended at 2 × 10^7^ cells/mL. Subsequently, each mouse was injected into the lower right flank with 100 μL of suspended cells. Tumor growth was examined every 3 days, and tumor volumes were measured as the length×width^2^×0.5. At 12 days post-injection, mice were sacrificed by CO_2_ asphyxiation, and the growth of each tumor was examined.

### Subcellular fractionation location

The separation of nuclear and cytosolic fractions was performed using the PARIS Kit (Life Technologies) according to the manufacturer's instructions.

### RNA immunoprecipitation (RIP)

RNA immunoprecipitation was used to investigate whether LL22NC03-N64E9.1 could interact or bind with the potential binding protein (EZH2, SUZ12 and LSD1.) in SW480 cells. We used the EZMagna RIP kit (Millipore, Billerica, MA, USA) following the manufacturer's protocol. SW480 cells were lysed in complete RIP lysis buffer, and the extract was incubated with magnetic beads conjugated with antibodies that recognized EZH2, SUZ12, LSD1 or control IgG (millipore) for 6h at 4°C. Then, the beads were washed and incubated with Proteinase K to remove proteins. Finally, purified RNA was subjected to qRT-PCR analysis to demonstrate the presence of LL22NC03-N64E9.1 using specific primers.

### Chromatin immunoprecipitation (ChIP)

Colorectal cancer cells were treated with formaldehyde and incubated for 10 mins to generate DNA-protein cross-links. Cell lysates were then sonicated to generate chromatin fragments of 200-300 bp and immunoprecipitated with H3K27me3, EZH2 and IgG as control. Precipitated chromatin DNA was recovered and analyzed by qRT-PCR. The primer sequences used for the studies are shown in Supplementary Table 1.

### Western blot assay

Protein was extracted from transfected SW480 cells and quantified as previously described using 12% polyacrylamide gradient SDS gel. Anti-GAPDH and anti-KLF2 were from Abcam (Hong Kong, China). ECL chromogenic substrate were quantified by densitometry (Quantity One software; Bio-Rad) while GAPDH antibody was used as control.

### Immunohistochemistry (IHC)

Xenograft tumor tissue samples were immunostained for H&E and Ki67. Anti-Ki67 was from Santa Cruz Biotechnology (Dallas, TX, USA). The IHC staining results were independently scored and compared by the author and a pathologist.

### Statistical analysis

All statistical analyses were performed using SPSS software version 22.0 (SPSS, Chicago, IL, USA). A paired, two-tailed Student's *t*-test or a chi-square test was used to evaluate significant differences between groups of data. All data are represented as means ± SD. Differences were considered significant if *P* < 0.05. “*” indicates P<0.05; “**” indicates P<0.01.

## SUPPLEMENTARY MATERIALS TABLE


