# Supplementary material for: A novel lncRNA, LL22NC03-N64E9.1, represses KLF2 transcription through binding with EZH2 in colorectal cancer
Source: Oncotarget. 2017 Jul 31;8(35):59435–45. doi: 10.18632/oncotarget.19738 (PMC5601744; doi:10.18632/oncotarget.19738)
Supplement: Supplementary file 1 [file oncotarget-08-59435-s001.pdf]

# A novel lncRNA, LL22NC03-N64E9.1, represses KLF2 transcription through binding with EZH2 in colorectal cancer

## SUPPLEMENTARY MATERIALS

**Supplementary Table 1: Sequences of primers for qPCR and siRNA sequence**

| Primers for qRT-PCR | Sequences (5' to 3') |
|---|---|
| LL22NC03-N64E9.1 (Forward) | AAGCCATGTAAAGGGGGCTC |
| LL22NC03-N64E9.1 (Reverse) | TGGTAGTCTGACCATTCTGCAT |
| GAPDH (Forward) | GAAGAGAGAGACCCTCACGCTG |
| GAPDH (Reverse) | ACTGTGAGGAGGGGAGATTCAGT |
| KLF2 (Forward) | CTGCACATGAAACGGCACAT |
| KLF2 (Reverse) | CAGTCACAGTTTGGGAGGGG |
| P15 (Forward) | GGACTAGTGGAGAAGGTGCG |
| P15 (Reverse) | GGGCGCTGCCCATCATCATG |
| LSD1 (Forward) | AGCGTCATGGTCTTATCAA |
| LSD1 (Reverse) | GAAATGTGGCAACTCGTC |
| EZH2 (Forward) | TGCACATCCTGACTTCTGTG |
| EZH2 (Reverse) | AAGGGCATTCACCAACTCC |
| P21 (Forward) | AAGTCAGTTCCTTGTGGAGCC |
| P21 (Reverse) | GGTTCTGACGGACATCCCCA |
| PTEN (Forward) | TGGATTCGACTTAGACTTGACCT |
| PTEN (Reverse) | GGTGGGTTATGGTCTTCAAAAGG |
| **Interference sequences (siRNA)** | |
| siRNA 1# (LL22NC03-N64E9.1) | UAGCUGGAGCAGUACAUCUUCAAUU |
| siRNA 2# (LL22NC03-N64E9.1) | AAGUAAUCAAGUUCCUGCUUUCUUG |
| shRNA-LL22NC03-N64E9.1 | TCTCAACATCTCTTCTTAATT |
| si-NC | UUCUCCGAACGUGUCACGUTT |
| si-EZH2 | GAGGUUCAGACGAGCUGAUUU |
